# The Role of Nutrition Across Production Stages to Improve Sow Longevity

**DOI:** 10.3390/ani15020189

**Published:** 2025-01-12

**Authors:** Matheus Saliba Monteiro, Rafaella Fernandes Carnevale, Bruno Bracco Donatelli Muro, Ana Lígia Braga Mezzina, Bruno Braga Carnino, André Pegoraro Poor, Carlos Emilio Cabrera Matajira, Cesar Augusto Pospissil Garbossa

**Affiliations:** 1 Nerthus Research and Development LTDA, Sao Carlos 13563-651, Sao Paulo, Brazil; matheus.saliba.monteiro@alumni.usp.br (M.S.M.); bruno.muro@poulpharm.com (B.B.D.M.); 2 Department of Animal Nutrition and Production, School of Veterinary Medicine and Animal Sciences, University of São Paulo (USP), Campus Pirassununga, Pirassununga 13635-900, Sao Paulo, Brazil; rafaella.carnevale@usp.br (R.F.C.); anamezzina@usp.br (A.L.B.M.); bruno.carnino@usp.br (B.B.C.); 3 Department of Veterinary and Biosciences, Faculty of Veterinary Medicine, Ghent University, 9820 Ghent, Belgium; 4 PoulPharm, 8870 Izegem, Belgium; andre.pegoraropoor@poulpharm.com; 5 Department of Biochemical and Pharmaceutical Technology, School of Pharmaceutical Sciences, University of São Paulo (USP), Sao Paulo 05508-000, Sao Paulo, Brazil; k.rlos89.cabrera@gmail.com

**Keywords:** fiber, growth rate, heat stress, microbiota, mortality, swine, peripartum

## Abstract

Modern hyperprolific sows are increasingly susceptible to health challenges. Their rapid growth rates predispose them to locomotor disorders, while high metabolic demands, reduced backfat thickness, and increased protein accretion heighten their vulnerability to heat stress and dystocia. Additionally, prolonged farrowing negatively affects the oxidative and inflammatory status of these females. Additionally, prevalent conditions such as gastric ulcers and cystitis raise ethical, welfare, and economic concerns. Despite the several studies related to sow nutrition, there are no studies which compile and extrapolate nutrition approaches from the rearing period and their impact on sows’ health and longevity. Also, the aim of our review was to shed light on gaps that require further investigation. Controlling body condition scores is crucial for maximizing productivity in sows. During gestation, high-fiber diets help maintain optimal body condition and prevent constipation, particularly during the peripartum period. Antioxidants offer a range of beneficial effects during this critical phase. Additionally, probiotics and acidifiers can enhance gut health and lower the risk of genitourinary infections. On the day of farrowing, energy supplementation emerges as a promising strategy to reduce farrowing duration. Collectively, these strategies address major health challenges, enhancing welfare and promoting sow’s longevity.

## Simple Summary

Over recent decades, the swine industry has achieved substantial productivity gains through advancements in genetics, nutrition, and management practices. However, at the same time, there has been a concerning rise in the number of sows being culled earlier than expected and an increase in sow mortality rates. The longevity of sows is crucial for sustainable pig production as it improves productivity and reduces costs. This early removal raises ethical and welfare concerns. Several factors influence sow longevity, making nutritional strategies critical for addressing these challenges and improving sow health and welfare. This article highlights key nutritional approaches to support sow development and longevity while identifying information gaps that warrant further research. For instance, young female pigs (gilts) should be fed separately from other pigs to ensure proper growth and bone development. Special diets are particularly important around the time sows give birth, as this is when they face the highest health risks. The use of fiber, probiotics or antioxidants can help improve the health and resilience of sows during these critical stages. These approaches are promising nutritional tools to improve sow health and welfare and can also benefit the industry’s profitability.

## 1. Introduction

Over the last few decades, the swine industry has significantly increased productivity through advancements in genetics, nutrition, and management [1]. Despite the significant boost in production, sows’ culling and mortality rates worldwide are increasing, affecting profitability and raising ethical and welfare concerns [2,3,4,5,6].

An optimal sow replacement rate is estimated to be around 40% for economical sustainability. However, industry data show a culling rate of 62% [5], with 47% of sows culled before their third farrowing, and nearly one-third of these removed as primiparous sows [7,8]. Furthermore, many producers cull sows before they reach their productive peak, which typically occurs between the third and fifth parities [9]. Health issues increase involuntary culling and mortality, and thereby, they limit the implementation of culling strategies based on reproductive performance. Ultimately, they reduce the overall profitability of the system and jeopardize animal welfare [10].

**Figure 1:**
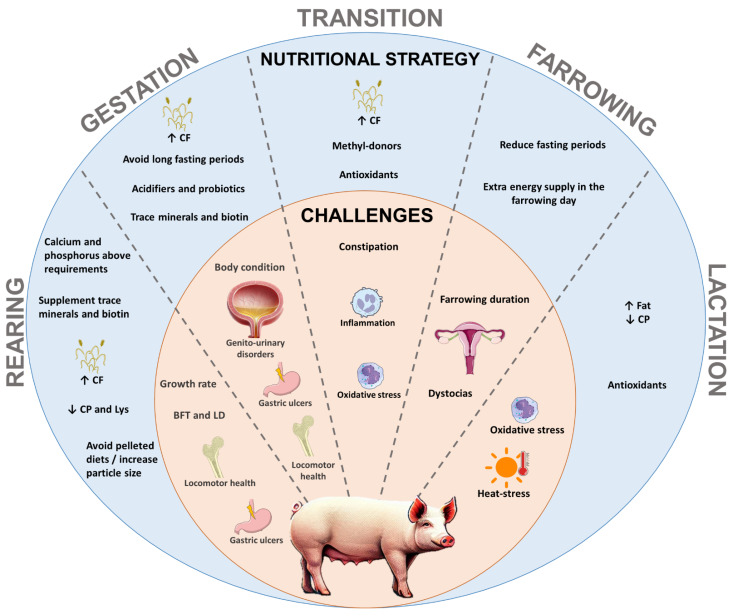
Main challenges affecting sow longevity across production stages and nutritional approaches to enhance health. This figure illustrates the major challenges influencing sow lifespan, including constipation, body condition score, BFT and LD parameters, inflammation, oxidative stress, dystocias, heat stress, genitourinary disorders, gastric ulcers, and locomotor health problems. It outlines targeted nutritional strategies for different stages—rearing, gestation, transition, farrowing, and lactation. Recommendations include adjusting CF, CP, and Lys levels, supplementing diets with acidifiers, probiotics, antioxidants, and trace minerals, and altering feed management according to the different production stages. CF: crude fiber; CP: crude protein; Lys: lysine; BFT: backfat thickness; LD: loin depth.

Not only health issues influence sow longevity; poor body condition scores or environmental conditions such as heat can decrease the lifespan of the sow [11,12]. The challenges in optimizing sows’ longevity by productive stage are presented in Figure 1. Many strategies can be used to tackle these issues, with nutrition being one of them. Traditional nutritional approaches have primarily focused on energy and protein density during gestation [13] and, more recently, on fiber inclusion [14]. These strategies’ main aim is to enhance productivity, particularly by increasing milk yield [15], improve the number of live-born piglets [16], and enhance piglets’ birth weight [17]. Modern approaches, however, emphasize not only productivity but also sow health and longevity [5], alongside the use of specific nutritional additives targeting microbiome modulation [18].

Based on this, the aim of this review is to explore nutritional strategies designed to improve sow health and longevity while enhancing reproductive performance, overall productivity, and the sustainability of the pig industry. Although nutritional requirements are fundamental to sows’ health, these are detailed in nutritional manuals and can vary significantly depending on factors such as environmental temperature, stress conditions, productivity levels, and genetic lines [17,19]. Therefore, determining specific nutrient levels is beyond the scope of this review. Instead, this review focuses on adaptable nutritional strategies that address challenges faced by different herds. Additionally, it highlights innovative nutritional approaches and identifies critical information gaps requiring further research. These strategies are not strict recommendations but can be tailored to specific situations. It is essential to emphasize that these are supplementary strategies and should be implemented in alignment with the requirements outlined in nutritional manuals.

## 2. Gilt Rearing Period

The successful development of a gilt begins even before its birth, as it is influenced by epigenetic effects and requires proper management of the dam [20]. Inadequate maternal nutrition alters the expression of genes associated with adipogenesis (SFRP2, SETD8, GCR, PPARγ, CCAAT, CEBPα, and FABP4) and litter size (RBP4) [21,22,23,24] of its offspring. Moreover, it also lowers birth weight, which is an important factor in determining longevity and productivity [11,25]. For example, gilts born weighing more than 1.28 kg have higher retention rates compared to those weighing less [11].

Early-life nutrition, particularly adequate colostrum intake, plays a crucial role in maximizing weaning weight and overall health [25]. Research indicates that inadequate colostrum intake at birth negatively impacts uterine gland development and future reproductive performance [26]. Modern hyperprolific lineages are selected for larger litter sizes, which in turn reduce birth weight, increase within-litter weight heterogeneity, and heighten suckling competition. These factors can hinder piglets’ colostrum and milk intake, ultimately impacting the future performance of gilts [11,25,27].

This section discusses nutritional strategies designed to better prepare gilts for the start of their reproductive life. Factors such as growth rate, weight at first mating, and feet and limb health are heavily influenced by rearing-period nutrition and can be managed to enhance long-term longevity.

### 2.1. Control of Growth Rate

Birth weight is positively correlated with sow longevity [28]. A cut-off point of 1 kg has been identified for selecting gilts to enter the herd to ensure adequate productivity and longevity [11,28]. To promote optimal health during the suckling period and enhance post-weaning adaptability, piglets should consume at least 250 g of colostrum, be weaned at a minimum of 24 days of age, and reach a weaning weight of at least 5 kg [11]. However, during the nursery phase, there is a notable gap in the literature regarding specific nutritional strategies for replacement gilts and the role of nutrition in their development. On the other hand, during the growing and finishing phases, it is known that gilts should not be raised as fattening pigs in mixed-sex groups until they reach reproductive age [24,29]. When gilts are fed standard growing and finishing diets, they tend to accumulate higher-than-ideal percentages of muscle mass for breeding purposes. Excessive muscle mass imposes an undesirable metabolic cost, as it demands additional energy intake for maintenance [30,31,32].

Dietary amino acid supply is a critical limiting factor for protein deposition, which directly impacts the average daily gain (ADG) of pigs. When dietary protein supply is reduced while maintaining the same energy level, fat deposition is favored [33,34]. Therefore, balancing protein and energy intake is essential during gilt development. Feeding trials with gilts provided diets containing varying crude protein (CP) concentrations (10.5%, 11.9%, 12.5%, or 15.4%) and two distinct energy levels have demonstrated this balance’s importance. Diets with reduced protein and high energy intake resulted in gilts with lower body weight, higher body fat percentage, and decreased lean tissue deposition [35].

While controlling the growth rate is crucial for maximizing longevity, it must not compromise the gilt’s overall development. Mammogenesis, for instance, begins around 90 days of age and continues until puberty [36]. Feed restriction during this critical period can reduce mammary parenchymal mass [37,38]. Studies suggest that lowering CP from 18.7% to 14.4% does not impair mammogenesis and helps maintain an adequate growth rate [37].

Fiber inclusion is another effective nutritional strategy to reduce energy density and restrict feed intake [39]. Gregory et al. [40] reported that diets containing 20.6% NDF (neutral detergent fiber) and 9.84% ADF (acid detergent fiber) reduced average daily gain (ADG) by 6%, resulting in lower body weight (BW) and backfat thickness (BFT) at mating. The authors found that this did not adversely affect mammogenesis, as gilts’ lactation performance remained uncompromised. Furthermore, the gilts in the high-fiber group tended to have reduced BFT loss during lactation [40].

Based on the aforementioned studies, a successful replacement gilt should have a birth weight of at least 1 kg, a weaning weight of at least 5 kg, and an average daily gain (ADG) of 400 g during the nursery period. From birth to mating, nutritional management should aim to balance the growth curve, maintaining an optimal ADG of 600–800 g to support longevity and reproductive efficiency.

### 2.2. Gilts’ Mating Weight

Weight at first mating is an important predictor of gilts’ longevity and future profitability. Some producers might favor mating heavier gilts due to the potential for larger first-parity litters. It was reported that litter size increases with higher mating weights, with an additional 0.4 piglets born for every 10 kg above 140 kg [41]. However, higher weight at mating and larger litters are also associated with a higher incidence of stillbirths and an increased number of piglets weighing less than 1.2 kg at birth [42]. First-parity females with a mating weight exceeding 170 kg are associated with negative outcomes in their second parity [31,43], including lower retention rates [44]. Mating heavier gilts may provide short-term gains but compromises long-term productivity. Conversely, light gilts (weighing less than 130 kg) produce fewer total piglets over the subsequent three parities [45].

Weight at first mating also influences long-term sow nutrition and compromises gilt feed efficiency. Gilts mated at 170 kg, as opposed to 140 kg for example, have their daily energy maintenance requirement increased by roughly 0.2 kg of feed daily [31]. At first mating, it is recommended that gilts are at least 180 days of age, have a BW ranging from 135 kg to 160 kg, and be on their second or third estrus in order to maximize longevity [11].

### 2.3. The Role of Micro- and Macrominerals and Vitamins in Limb Health

Ensuring adequate nutritional intake of minerals during gilts’ development is crucial since these elements are mobilized during high nutritional demand. Mahan [46] describes that sows prioritize fetal development and lactation maintenance at the expense of their own body mineral reserves. Consequently, gilts that have low calcium (Ca) and phosphorus (P) reserves tend to have health issues such as reduced bone size and strength, growth impairment, lameness, and osteochondrosis [5,46,47,48].

Sows’ foot and leg health is critical for longevity as they are expected to farrow over two litters per year living on concrete floors [49]. From 12 to 20% of culls happen on account of leg/hoof disorders [50,51]. Lameness and other locomotor problems are also major causes of euthanasia, with locomotor lesions being reported in more than 70% of necropsied sows [12,52,53]. Kirk et al. [52] found that arthrosis or osteochondrosis represented over 88% of all sows’ post-mortem findings. These data raise animal welfare concerns and demonstrate the high impact of locomotor disorders on herd profitability. Considering sows’ high demand for minerals during reproductive cycles, it is suggested that Ca and P supplementation during gilt development should be above the levels suggested by the NRC [5,19]. Specifically, it has been suggested that increasing NRC levels by 8% maximizes bone mineralization and growth rate [11,19,54].

Hoof lesions are highly prevalent in sows and may lead to other locomotor disorders due to ascending infections [12]. Lameness and hoof lesions are reported to be reduced when trace minerals like zinc (Zn), manganese (Mn), and copper (Cu) are supplemented in the diet, due to their importance on hoof structure and integrity [55,56,57,58]. An inadequate supply of trace minerals may result in low-strength corneal tissues, making pigs prone to locomotor disorders [59]. These minerals are also related to metalloproteinases and growth factors that are essential for the renewal of the extracellular matrix in bone and connective tissue, also described as important to avoid lameness [60,61].

Incorporation of chelated trace minerals, which likely have higher bioavailability, has shown promising results in improving gilts’ hoof health [58,62,63]. Supplementing chelated trace minerals and methionine during the rearing period reduces gilts’ lameness and improves their performance [62]. Da Silva et al. [64] described an improvement in pigs’ hoof integrity when chelated minerals Zn, Mn, and Cu (50 mg/kg, 20 mg/kg, and 10 mg/kg, respectively) were supplemented during the finishing phase. The supplementation reduced the incidence of dew claw damage and heel-sole cracks by 22.02% and 21.26%, respectively. Similar findings were observed when sows were supplemented with minerals chelated with methionine (50 mg/kg Zn–methionine, 20 mg/kg Mn–methionine, and 10 mg/kg Cu–methionine) [65].

Vitamin intake, particularly vitamin D and biotin, plays a significant role in limb health, especially in bone structure [59]. Biotin is essential for the keratinization process to ensure horn integrity [59] and is also required for the production of intercellular cementing substance (ICS) and complex lipid molecules [66]. Vitamin D is crucial for calcium (Ca) and phosphorus (P) metabolism and bone remodeling [59].

In dairy cows, a meta-analysis indicated that biotin supplementation improves hoof health [67]. However, results for sows are controversial. These discrepancies may stem from differences in the amount and duration of supplementation, biotin bioavailability, the biotin status at the start of the study, and the age and parity of the sows [59]. To heal claw lesions, dietary biotin concentrations exceeding 200 ng/kg of dry matter are necessary [68]. Supplementing 350 μg/kg of biotin in the feed for female pigs from 25 kg body weight until the weaning of their first litter reduced hoof lesions [69]. Another study found that gilts supplemented with biotin at 1160 μg/day throughout gestation and lactation had fewer heel lesions [70]. Despite these promising results, most studies evaluating the impact of biotin supplementation on sows’ hoof health are outdated. Updated research is needed to assess the effects of different biotin doses in modern genotypes and to explore their interactions with group-housing systems.

Understanding vitamin D supplementation requires careful consideration of its complex interactions with Ca and P [71]. An inadequate balance among these factors may impede bone mineralization and strength [72]. For instance, increasing dietary Ca levels from 6.45/6.85 to 8.56/8.99 g/kg in gilts weighing 30 to 180 kg was reported to negatively impact phosphorus utilization [71]. For pigs, accurate reference values for evaluating vitamin D status at different ages and reproductive stages are still lacking [73]. Additionally, factors such as housing systems may influence vitamin D requirements or the need for more biologically active forms of supplementation. Pigs raised outdoors or in confinement with access to sunlight have higher plasma concentrations of 25-hydroxycholecalciferol (25-OHD3) [73,74], which can positively impact bone health and reduce lameness. Future studies should focus on clarifying the efficiency of vitamin D metabolite absorption from the diet and determining the optimal plasma levels required for high-performing replacement gilts.

### 2.4. Particle Size of Diets

Swine species are particularly susceptible to the development of gastric ulcers, primarily due to the vulnerability of the aglandular *pars oesophagea* [75]. Some studies reported that more than half of necropsied sows had gastric ulcers [12,52,76,77], and hemorrhage secondary to gastric ulcers has been identified as an important cause of sows’ mortality [12]. Moreover, parakeratosis in the *pars oesophagea*, a chronic condition, is a common post-mortem finding. The gastric ulcer develops during piglets’ early life (after weaning), and even after healing, it can be easily reactivated [78].

Several risk factors predispose pigs to gastric ulcer development [78], but among the nutritional aspects, two are particularly noteworthy: feed particle size and pellets. Ayles et al. [79] reported that a diet with a mean geometric particle size of 578 µm increased the severity of gastric ulcers, reduced the feed intake, and worsened the feed/gain ratio when compared with pigs fed a coarser diet (937 µm). Similar results have been reported by Wondra et al. [80]. Another study identified that feeding a pelleted diet is a catalyst for gastric ulcers [81]. While pelleting and reduced particle size can improve ADG and feed conversion in finishing pigs [80,82], they can compromise gilts’ longevity.

Gilts require controlled growth, and thus, the use of fibers and coarser diets can be beneficial by not only controlling growth but also reducing gastric ulcer prevalence [75,81,82,83]. Furthermore, a temporary change to a mash feed for a short period might help to heal ulcers without a significant loss in feed efficiency [79]. Although few studies have evaluated the effects of mean particle size on the development of gastric ulcers in sows, a diet with a mean particle size between 750 and 1200 µm may be beneficial [82]. The choice of an optimal particle size should also take into account the incidence of gastric ulcers observed in the herd, as well as the impact of other nutritional strategies, such as fiber inclusion. Other nutritional factors during the rearing period discussed in the text are summarized in Table 1.

## 3. Gestation Period

The majority of a sow’s productive lifespan occurs during gestation. Inadequate feeding management and diet composition can affect metabolic processes and reduce sow longevity. In contrast, ensuring an adequate body composition throughout gestation optimizes performance during farrowing and lactation [24]. It is during this period that high cystitis incidence is observed, which can contribute to increased mortality [12] and endometritis [87].

Another key aspect regarding sows’ longevity is the trend for group housing [88]. Group housing sows increases aggression behaviors and lameness [89] and can have detrimental impacts on sows’ body condition score (BCS) [88]. All these aspects can negatively impact sows’ health and consequently longevity. Nutritional and feeding management strategies are useful to deal with these issues, maximize sow welfare, and thus increase longevity, which will be further explored in the following section.

### 3.1. Body Condition Score

Studies have indicated that sows with a low BCS are at higher risk of death [52,90]. Additionally, reduced energy intake during gestation has been linked to higher culling rates [91] and reduced BFT and loin depth, both compromising longevity [24,92]. Sows on the other extreme of BCS, namely obese sows, have an increased risk of mortality due to heart failure [12,93]; thus, BCS must be carefully controlled in order to maximize sow longevity.

BCS should be managed to achieve optimal levels throughout gestation, with early gestation (first 30 days) identified as the best period for sows to recover body reserves [94]. Previously, it was believed that increasing feed intake during this phase could elevate progesterone metabolism and lead to higher embryo mortality due to excessive energy supply. However, a recent systematic review by Leal et al. [95] refuted these concerns, indicating that energy intakes as high as 12,900 Kcal ME/day did not affect embryo survival. Supporting this, Athorn et al. [96] reported that feed restriction during early pregnancy reduced progesterone levels and embryo numbers, highlighting the importance of sufficient nutrient intake during this critical phase.

Diets aimed at recovering BCS should include higher CP levels than maintenance diets, particularly for younger sows. Johannsen et al. [97] demonstrated that diets containing 3.37 to 6.39 g of standardized ileal digestible (SID) Lys/kg promoted a linear weight gain, associated with higher protein accretion, without any reductions in litter size or piglets’ birth weight. This approach was markedly effective in restoring BCS for second- and third-parity sows, compared to older sows (fourth and fifth parities). During mid-gestation, body weight gain showed a quadratic response to SID Lys levels, peaking at 5.0 g SID Lys/kg diet [97].

For the transition period, sows should be fed 5.79 g SID Lys/kg to ensure a high milk yield in the following lactation [98]. It is important to emphasize that these requirements were estimated for hyperprolific Danish sow lines, with an average total of 21 piglets born per litter.

On the opposite end of the BCS spectrum, heavy sows present their own set of challenges. Obesity leads to metabolic disorders, a persistent pro-inflammatory state, and insulin resistance [24]. During the peripartum period, these metabolic changes exacerbate oxidative stress, which can result in dystocia and prolonged farrowing [24]. In lactation, obese sows face higher catabolism and reduced feed intake, leading to significant weight loss and a lower BCS in the subsequent cycle [24]. This combination of elevated catabolism and low post-lactation BCS has been linked to poorer reproductive performance in subsequent cycles [10,24,99].

Feeding management should be performed according to BCS, parity order, and genetic line, and increasing SID Lys can help recover the body condition of younger sows, especially considering precision nutrition. In addition, for sows that finish lactating with a low BCS, employing strategies such as skipping a heat cycle or using altrenogest prior to weaning, prolonging the weaning-to-estrus interval to allow body condition recovery, are effective interventions to improve sow longevity and reproductive performance [100,101].

BFT is a key objective measure of fat reserves, closely associated with BCS. Ideally, it should be maintained within the range of 15 to 20 mm [24]. In addition to fat deposits, muscle deposits play a crucial role. However, the limited number of studies examining loin muscle depth and area, as well as their impact on sow longevity, prevents the establishment of definitive range recommendations [24].

To manage body composition, gilts and sows in optimal body condition should receive 6650–7000 and 6800–7900 kcal per day, respectively [24]. Parity 1 and 2 hyperprolific sows with a low BCS should be fed 17 g/day of SID lysine during early gestation to maximize BW gain [97]. During late gestation, sows should be provided with 22 g/day of SID lysine to optimize lactation performance [98]. Additionally, the inclusion of functional amino acids, particularly arginine or its precursors, has the potential to enhance sow performance without the need for overfeeding. This approach can positively influence sows’ longevity by supporting their overall health and productivity [102].

### 3.2. Feeding Frequency

Historically, sows are fed only once per day, to ease the farm’s routine. However, this feed regimen increases hunger-related behaviors and activates the hypothalamic–pituitary–adrenal axis, both negatively impacting the metabolism and welfare [103,104,105,106]. Feeding sows once a day is also associated with a higher incidence of gastric ulcers and abdominal organ torsion, increasing sows’ mortality [52,107,108]. This practice disrupts digestion kinetics and hormone secretion (insulin, ghrelin), and lowers blood glucose and IGF-1, which in turn impacts overall metabolism [104,106]. Longer fasting periods before farrowing increase farrowing duration [109], consequently impairing sows’ health [110].

### 3.3. Feed Management in Group Housing

There is a global trend towards group housing for sows to improve animal socialization and welfare [88]. However, group housing poses a challenge for the nutritional management of sows. As hierarchical animals, pigs often display aggressive behavior during feeding times [111,112]. To address these challenges, individualized nutrition tailored to each sow’s BW, fat reserves, and muscle composition is essential for promoting longevity. Achieving this, however, requires appropriate feeding systems and housing strategies to mitigate the negative effects of group housing

There are three main feeding strategies in group-housing systems: floor feeding, partial stalls, and electronic sow feeders (ESFs) [88,112]. Floor feeding is the simplest and cheapest strategy, and has the upside of fulfilling some of the sow’s natural feeding behavior [112]. In this strategy, sows receive the feed directly on the floor and are not separated from each other by any structure. The downside of floor feeding is that sows’ feed intake can be influenced by the hierarchical structure within the group. After being group housed, sows fight to form a hierarchy; dominant sows have feeding rights preference, and thus can consume subordinate sows’ daily feed amount. This leads to a variation in feed consumption between dominants and subordinates, resulting in over- and undernutrition, respectively [113]. Additionally, gilts are slow eaters, and systems without protection during feeding or those that do not separate sows by parity can lead to undernourishment of the younger females [114].

Partial stall feeding is similar to floor feeding, where sows receive feed directly on the floor. However, aggression is reduced as sows are partially separated by railings during feeding. Both of these feeding strategies are less precise in group housing compared to ESF systems, which allow for individualized feeding regimens [112]. ESF systems adjust feed curves based on factors such as the sow’s BCS [112], parity, and gestation stage, enabling more precise nutrition management.

Despite these advantages, ESF systems also raise welfare concerns [88]. In natural settings, pigs eat in social groups simultaneously rather than individually and sequentially [88]. In ESF systems, sows form feeding queues since each station accommodates only one sow at a time. This queuing increases stress and may impair gastrointestinal physiology [115], contributing to the development of gastric ulcers. While reducing feeding time in ESF systems has been proposed as a solution, this approach did not decrease queuing and led to more sows consuming less than the stipulated feed amount [88,114].

An alternative is changing diet composition to include a higher fiber content. While high-fiber diets increase the time spent in the feeder [88,115], they also increase satiety [115,116] and prevent gastric ulcers [75,83]. To address aggression, the inclusion of tryptophan (TRP) in the diet seems to be a promising approach. TRP is a precursor of serotonin (5-HT), a neurotransmitter capable of reducing aggressive behavior and responsible for regulating sleep, appetite, and mood [117,118,119,120]. TRP supplementation for seven days at three times the standard requirements increased blood TRP and serotonin concentrations, and reduced aggressiveness in growing gilts [121]. In another study, this effect was consistent for gestating sows, when tryptophan was included at a 220% level [122].

### 3.4. Probiotics Supplementation

Most swine studies on probiotics focus on the microbiome modulation, intestinal health, and productivity of piglets [18]. Few studies have investigated the impact of probiotic supplementation on sows’ health [18]. Despite this, the gastrointestinal (GIT) microbiome of sows significantly impacts gut permeability and plasma endotoxin absorption, contributing to metabolic disorders and a pro-inflammatory state during early lactation [123]. Additionally, it plays a critical role in the development of urogenital and mammary diseases [18,87]. Probiotics not only favorably modulate the intestinal microbiome but may also decrease plasma metabolites such as ammonia (AMM), total cholesterol (TC) and low-density lipoprotein cholesterol (LDL-C), and inflammatory markers (IL-1β, IL-2, IL-6, and IFN-α) [124]. This effect is particularly relevant during the postpartum period, as it plays a pivotal role in maintaining overall health and enhancing reproductive performance [124,125]. Moreover, the gut microbiome may potentially influence resistance and modulate the immune response to other extraintestinal pathogens, such as PRRSv [126,127]. This is particularly important since PRRSv-positive herds are reported to have a higher mortality risk [3].

Specific probiotic strains of *Lactobacillus* spp. and *Bacillus* spp. have been reported to prevent pathogenic colonization, reduce inflammation, and maintain the physiological balance of the intestinal mucosa during critical gestational stages [128,129,130]. *Enterococcus faecium* supplementation during the transition period and lactation also improved sows’ BCS [131]. Moreover, piglets born from probiotic-supplemented sows seem to be more resistant to an *E. coli* K88+ challenge, produce less pro-inflammatory cytokines, and have reduced amounts of the *Clostridium* and *Brachyspira* genera while increasing Lactobacilli presence in their microbiome [132]. *Bacillus* spp. supplementation reduces catabolism during lactation, improves sows’ diet digestibility, and enhances piglet performance during the suckling period [132,133,134]. Furthermore, *Clostridium butyricum* supplementation has been reported to improve farrowing kinetics and piglet performance [135].

Chronic stress and elevated cortisol levels impair sow metabolism and intestinal integrity, adversely affecting longevity [136,137]. Although the brain–gut microbiome axis has been extensively studied in other species, its role in pigs remains underexplored [138,139]. Pereira et al. [140] indicated that supplementation of a multistrain probiotic (*Lactobacillus* spp.—four different species, *Enterococcus faecium*, *Bifidobacterium bifidium*, and *Streptococcus thermophilus*) throughout gestation and lactation resulted in sows showing less fear of human interaction. Cortisol levels were halved and serotonin levels increased by 11% compared to non-supplemented sows. These findings suggest that by modulating only the microbiome part of the axis, the neurological and behavioral health of sows could be altered, lowering aggressive behavior and improving longevity.

Several biomarkers linked to productivity and disease have been identified, but further research across diverse herds is needed to establish causality and validate these findings. Future investigations should prioritize systems biology approaches to examine microbial–microbial and microbial–host interactions, as well as the impact of microbial metabolic products on reproductive performance and disease incidence. Randomized, blinded clinical trials will be essential to determine whether targeted modulation of microbial genera identified as biomarkers in metagenomic studies can positively influence reproductive outcomes and disease management.

### 3.5. Acidifiers

Dietary acidifiers have become a focal point in recent scientific research, with promising evidence for improving animal health and reducing the need for antimicrobials [141]. Dietary acidifiers include organic and inorganic acids, acid salts, and acid blends, which may be administered through drinking water or feed [142,143,144].

Inorganic acids contain a non-metal element; they are often used as feed additives due to their lower cost compared to organic acids. Examples include hydrochloric acid (HCl), phosphoric acid (H_3_PO_4_), and sulfuric acid (H_2_SO_4_) [145]. Organic acids (OAs) are the most commonly added acid in swine diets [144], but their production by the animal can also be stimulated by the diet composition [146]. Organic acids can be categorized into three main types: short-chain fatty acids (SCFAs), medium-chain fatty acids (MCFAs), and tricarboxylic acids (TCAs) [147]. SCFA, MCAs, and TCAs are produced in the lower intestine through microbial fermentation. These acids are essential for maintaining intestinal health, reducing inflammation, and serving as an enteric energy source [148,149]. Other organic acids include sorbic, benzoic, and lactic acids.

Although the exact mode of action of organic acids is not yet fully understood, it is believed that they work mainly by inhibiting pathogenic microbes by lowering gut pH [150,151]. In addition, some SCFAs may exert antioxidant functions by regulating oxidoreductases [152,153]. Butyrate protects against H_2_O_2_-induced DNA damage in rats and humans [154,155], while acetic and butyric acids reduce oxidative stress in mesangial cells induced by high levels of glucose and lipopolysaccharides [156]. Furthermore, butyric acid has been identified as a regulator of the nuclear factor erythroid 2-related factor 2 (Nrf2), an essential transcription factor involved in the defense against oxidative stress [157].

Most of the studies related to acidifier supplementation focused on weaning piglets, but still, some reports can be found about its effects on sows. According to Devi et al. [158], sows fed organic acid diets had decreased *E. coli* and increased *Lactobacillus* content in feces during farrowing and the weaning period. This microbiota profile can reduce LPS absorption in the sow gut, reduce inflammation, and exert a positive effect on piglets’ gut colonization [159]. Wu et al. [151] reported that adding a probiotic and a blend of acidifiers to the diets of lactating sows reduced malondialdehyde (MDA) levels and increased superoxide dismutase (SOD) concentrations, indicating that the combination of probiotics and acidifiers can improve antioxidant function. Furthermore, the incorporation of 0.05% tributyrin, a butyrate alternative, during the last 35 days of gestation increased sows’ anti-inflammatory cytokines IL-6 and IL-10 and increased *Lactobacillaceae* in the intestines of piglets and sows [160]. Moreover, Lin et al. [160] observed that tributyrin supplementation reduced farrowing duration, which could contribute to reducing postpartum diseases and consequently increase longevity.

Moreover, acidifiers can lower urinary pH and decrease the risk of urinary tract infections (UTIs), which are an important cause of culling and mortality in sows [12]. In a study examining the electrolyte balance in sow urine, DeRouchey et al. [161] showed that a decrease in urinary pH reduced total bacterial concentrations in urine, potentially lowering the risk of UTIs. Kluge et al. [162] demonstrated that the addition of 0.5%, 1.0%, and 2.0% benzoic acid to the diet led to a linear decrease in urinary pH compared to the control group. The reduction in *E. coli* excretion in feces in sows supplemented with OAs may favorably reduce genitourinary colonization by these bacteria and consequently reduce genitourinary diseases [163,164].

The use of dietary acidifiers can positively impact sow health, primarily by modulating the microbiome and reducing the excretion of potentially pathogenic bacteria in feces. Acidifiers may also help alleviate oxidative stress and inflammation during critical stages of a sow’s productive life, such as the peripartum period and lactation. Moreover, the increasing antimicrobial resistance observed in *E. coli* isolated from genitourinary discharges [164] underscores the need for alternative strategies to prevent cystitis and metritis.

Despite these promising benefits, high levels of acidifiers can reduce diet palatability and feed intake [165], which must be considered when formulating diets. Additionally, traditional organic acids primarily affect stomach pH, with limited action in the gut [142]. In contrast, monoglycerides have better palatability [166] and are released only in the intestine, offering greater potential for microbiota modulation [167]. However, further research is needed to evaluate their impact on sow’s health.

While acidifiers alone can significantly improve health, combining them with other strategies—such as probiotics, prebiotics, or dietary fiber—may amplify their benefits, reducing antimicrobial use and enhancing sows’ health and longevity. Further studies are required to identify optimal blends and concentrations of organic acids, their interactions with other feed additives, and their effects on feed intake, cystitis prevalence, and sow longevity.

### 3.6. Crude Fiber and Sows’ Health

Incorporating dietary fiber into the diets of sows and gilts is an effective nutritional strategy for enhancing the sustainability of swine production [168]. Sourced from plant-based ingredients and by-products of the food and beverage industries, dietary fiber provides multiple benefits, including improved feed cost efficiency, reduced environmental impact, and enhanced animal welfare [169,170,171].

Fiber’s physiological effects are driven by its heterogeneous nature, with properties like solubility, viscosity, fermentability, and water-holding capacity influencing digestion, metabolism, and the gut microbiota. These attributes can regulate satiety, stabilize blood glucose levels, and promote appetite control, supporting overall health and resilience [14,172,173]. By leveraging these benefits, dietary fiber plays a pivotal role in supporting the longevity and productivity of sows in modern swine production systems [174,175,176].

Ingredients rich in soluble fiber exhibit superior hydration-related properties, such as enhanced water-holding capacity, bulking, and viscosity, compared to those rich in insoluble fiber. These characteristics play a significant role in stabilizing blood glucose levels, reducing interprandial glycemia fluctuations, and preventing insulin resistance during late gestation, thereby promoting metabolic stability and improving overall welfare [14,172,173]. Additionally, the higher the proportion of soluble fiber, the higher the capacity of the ingredient to be fermented in the hindgut to produce SCFAs [177]. SCFAs can provide up to 30% of a sow’s maintenance energy requirements, which is particularly beneficial during late gestation and lactation when energy demands are elevated [178]. Even in pre-mating gilts, including fiber in the diet for 14 days prior to artificial insemination has positive effects. It helps regulate glucose and IGF-1 metabolism, thereby enhancing follicular nutrition and development [179,180].

Several studies have indicated that fiber has the potential to reduce gastric ulcers [83,86,116]. However, few studies have evaluated the impact of different fibrous ingredients and their properties on the development of gastric ulcers [116]. One study reported that the addition of 5% whole sunflower hulls to basal diets containing 4–5% crude fiber (CF) reduced the incidence of severe gastric mucosal lesions by almost 50%. Despite this reduction, the study observed a high overall incidence of gastric lesions, with nearly 90% of animals developing mucosal damage [83]. Millet et al. [86] evaluated the effects of particle size and fiber content on piglets from weaning to slaughter. They observed that increasing the CF content from 3–4% to 8–9% reduced the incidence of gastric mucosal lesions from 93.5% to 53.2%. Importantly, the effects of fiber inclusion were more pronounced when the particle size increased from 500 to 700 μm [86]. High-fiber diets increase retention time in the stomach, resulting in earlier satiety and reducing the time that the *pars oesophagea* is exposed to gastric acid, which can help to prevent gastric ulcers [86]. It is essential not only to determine the optimal CF concentration, but also to understand the effects of different fiber types with varying retention times, bulking properties, and solubility. Based on the discussed evidence, the recommended inclusion of fiber to reduce gastric ulcers ranges from 5 to 10%. This inclusion should be optimized based on the animal’s age, the type of fiber, and the feed particle size, which is recommended to be between 750 and 1200 μm.

Ingredients rich in insoluble fibers are characterized by poor fermentability. Consequently, these ingredients contribute to a lower energy density, allowing nutritionists to dilute the energy content of diets while maintaining a reasonable feed volume [39,40]. This strategy helps manage sows’ BCS during gestation, promoting optimal weight maintenance and preventing excessive gain, while supporting satiety and overall welfare [24,181,182]. The incorporation of fiber into gestation diets to increase bulk has been shown to reduce stereotypic behaviors by 7–50% while also decreasing general restlessness and aggression [116]. Research indicates that a CF content of 10% positively affects behavior, while a CF content of 20% more significantly enhances short-term satiety, reduces postural changes, and increases resting time [183]. It is recommended that sows’ feed should contain at least 8% CF, or a daily intake of at least 200 g of CF per sow, up to one week prior to farrowing. This approach effectively reduces aggressive behaviors and improves welfare [184].

Moreover, insoluble dietary fiber plays a crucial role in stimulating intestinal transit, a particularly desirable characteristic during late gestation and the pre-farrowing period when sows and gilts are more susceptible to gastrointestinal constipation [185]. Pre-farrowing constipation is a common disorder in commercial swine operations that may affect up to 70% of the sows within a herd [186]. Constipation can lead to dysbiosis, which in turn triggers inflammatory and oxidative stress responses. This condition increases the absorption of LPS endotoxins, negatively impacting farrowing outcomes and compromising sow health [186]. Dumniem et al. [187] observed a significant reduction in constipation prevalence among parturient sows, decreasing from 46.3% to 17.6%, when a fibrous supplement increased the diet’s CF content from 4.3% to 5.5%. Similarly, Oliviero et al. [188] reported a reduced risk of constipation in sows when the dietary CF content was increased from 4% to 10%. These findings collectively underscore the effectiveness of dietary fiber supplementation during the transition phase in mitigating constipation in sows.

Despite a growing consensus on the benefits of dietary fiber for the health, welfare, and reproduction of pigs, its inclusion in the diet remains limited in several countries. Before expanding the incorporation of fibrous ingredients into sows’ diets, a thorough understanding of the physicochemical properties of each ingredient and their impact on gastrointestinal tract physiology and metabolism is essential. Both soluble and insoluble fibers have shown positive responses to intestinal health, systemic inflammatory profile, and glucose and lipid metabolism in pigs and humans [169,177,189]. It is argued that combining both types of fiber produces a greater response of parameters related to gut health [14,190,191], but more studies are needed to evaluate their combined effect on the welfare, performance, health, and metabolism of sows.

### 3.7. Bump Feeding

Bump feeding is a nutritional strategy in which feed intake is increased from approximately 90 days of gestation until parturition [102]. Initially adopted to enhance piglet birth weight—given that fetal growth is at its highest during this gestational phase —the effectiveness of bump feeding remains inconsistent. Some studies have reported benefits, such as increased birth weight [192,193] and increased milk yield [192], while others have found no significant effects [102]. In some cases, bump feeding has even been associated with negative outcomes, including higher stillbirth rates [193] and low sow feed intake during lactation [194].

Emerging research suggests that amino acid supplementation during late gestation may provide better productivity outcomes than simply increasing overall feed intake [102,195]. Bump feeding may also lead to overnutrition [24,196], which impairs sow longevity [24]. An effect of bump feeding that seems to be more consistent is the improvement in sows’ BCS [194]. This is noteworthy because low BCS is linked to increased mortality risk [52,90] and reduced longevity [3,24,196]. However, this also highlights that this strategy should be avoided in over-conditioned sows.

Pelvic organ prolapse is one of the main causes of sows’ death and mostly occurs during the peripartum phase, right after bump feeding’s recommended implementation period [12]. Although prolapse etiology is not fully understood, inadequate BCS appears to increase its incidence [197]. Thus, the potential positive effects of bump feeding may be linked to improvements in BCS, which warrants further investigation. However, it is important to emphasize that early gestation presents the optimal window for recovering BCS. Nutritional strategies, feed additives, and optimal BCS during gestation are presented in Table 2.

## 4. Peripartum—Transition Diet

The transition period is a critical period in a sow’s life. During this period, sows produce colostrum, initiate and maintain milk production, and undergo the farrowing process, shifting their metabolism from an anabolic to a catabolic state. Concomitantly, they are moved from gestation facilities to farrowing rooms and experience an inflammatory state and dietary changes [125]. This scenario increases their susceptibility to puerperal diseases, which can hinder their colostrum and milk yield [125]. Due to these factors, sows’ mortality rates during the transition period account for up to 50% of all deaths [12].

The primary goals during this phase are to reduce farrowing duration and prevent puerperal diseases. Prolonged farrowing impacts offspring’s vitality and colostrum yield and increases the risk of dystocia [199,200] and, consequently, the occurrence of metritis [12]. Metritis also negatively impacts milk yield and can further compromise the litter’s health and growth [18]. In addition, postpartum dysgalactia syndrome (PPDS) is a great concern for sow culling and has a multifactorial etiology. PPDS can affect approximately 13% of sows within a herd [201]. It is well known that nutritional strategies during the transition period can be used to reduce farrowing duration and reduce PPDS [125,200].

Based on the aforementioned, it is crucial to understand the physiology of transition sows to develop nutritional strategies that enable them to prepare and cope with the rapid and substantial increase in metabolic demand around farrowing. This, in turn, can improve sow longevity [202].

### 4.1. Negative Dietary Cation–Anion Difference (DCAD) Diets and Calcidiol Supplementation

The expulsion of fetuses is dependent on uterine contractions and determined by oxytocin release and its interaction with other hormones [203]. Along with oxytocin, serum calcium concentration plays a crucial role in myometrium contraction, influencing the frequency, intensity, and coordination of contractions [204]. Serum calcium concentration is finely regulated by parathyroid hormone (PTH), calcitonin, and vitamin D. These hormones regulate the absorption of calcium from the gut, its reabsorption in the kidneys, and mobilization from skeletal reserves [205].

The relationship between circulating free calcium and farrowing traits is complex. Data from other species suggest that suboptimal serum calcium concentration may prolong farrowing duration [203]. However, feeding prepartum sows with diets that exceed their calcium requirements compromises calcium homeostasis [206], and may compromise calcium reserve mobilization.

In dairy cows, a negative DCAD is commonly adopted during the transition period [207], typically achieved by including acidogenic protein meals in the ration [202]. DCAD reflects the difference between the milliequivalents of the principal cations (potassium [K] and sodium [Na]) and the principal anions (chloride [Cl] and sulfur [S]). A negative DCAD diet induces the release of hydrogen ions, enhances the sensitivity of osteoclasts to PTH, and increases the formation of calcitriol [208,209]. These hormones increase Ca turnover by reabsorbing Ca in the bones and increasing their absorption in the gut, which increases the supply that can be used during farrowing [210,211].

Some studies in pigs have reported positive effects of negative DCAD, such as reducing stillborn piglets and improving subsequent fertility rate [161,212,213,214]. Guo et al. [209] used corn–soybean meal control diets with calculated DCAD values of 170 mEq/kg during late gestation (from day 94) and 226 mEq/kg during lactation. Reducing the DCAD by 100 mEq/kg resulted in higher serum calcium levels on the first and eighteenth days of lactation [209]. Darriet et al. [215] supplemented the peripartum sows with an acidogenic mineral diet containing 17% Cl, 6.3% Mg, 2.5% Ca, 7.5% NH_4_, and 8.7% SO_4_ with levels ranging from 0 to 2.5%, and mEq values from 33 to −216 mEq. The study found that blood and urine pH decreased linearly while blood calcium increased proportionally to dietary acidogenic mineral supplementation [215]. In addition, some studies indicate that anionic diets may exert effects even in subsequent reproductive cycles [212,213].

Calcidiol supplementation has been linked to increased colostrum [216] and milk yield [216,217], reduced dystocia’s incidence [217], higher piglet birth weight [216], a lower incidence of fever [217], and fewer stillborn piglets in sows supplemented with 1400 IU/day or higher [72]. In another study, calcidiol supplementation associated with an anionic diet and administered from 104 days of gestation to 4 days after farrowing reduced stillbirth occurrence and increased the number of born-alive piglets in the subsequent farrowing [202].

Additionally, the reduction in urine pH associated with low-DCAD diets may have other positive effects, such as reducing *E. coli* proliferation and shedding, which could influence bladder and vaginal microbiomes and decrease cystitis prevalence [18]. Further studies are necessary to determine the ideal mEq in diets and their interaction with the use of low-calcium diets in preparturient sows. Moreover, negative DCAD might be used as a strategy to reduce urine pH in other gestation phases beyond peripartum to decrease cystitis prevalence. This approach could reduce antimicrobial use, and its impact on sow health and piglet development should be further studied.

### 4.2. Antioxidants

Lapointe [218] highlights in their review that modern sows have increased metabolic demands, placing significant stress on the mitochondria to meet these requirements. This is especially pronounced in the final third of gestation and lactation, periods with high metabolic demand and release of inflammatory mediators, leading to oxidative stress [219]. Mitochondrial activity generates a substantial amount of free radicals; both the performance and longevity of sows are linked to mitochondrial function. When reactive oxygen species (ROS) production exceeds the endogenous total antioxidant capacity, it can lead to lipid peroxidation, protein and DNA damage, and, ultimately, a shortened lifespan in sows [220,221,222]. DNA damage increases from 60 days of gestation throughout lactation, while antioxidant levels substantially drop after 110 days of gestation, not to be fully recovered until weaning [219].

Antioxidants are particularly valuable in counteracting the detrimental effects of ROS production, especially during the peripartum period. Plant-based ingredients are rich in CF and active compounds with reductive capacity, such as terpenes, phenols, flavonoids, proanthocyanidins, glycosides, saccharides, aldehydes, esters, alcohols, vitamin C, vitamin E, beta-carotene, zinc, and selenium [222,223]. These compounds act in different pathways, including microbiome modulation and the enhancement of antioxidant activity [224].

Several studies have evaluated the supplementation of plant-derived ingredients in the last third of gestation: *Pennisetum* (5 to 10%) [181], hemp seed (2%) [225], *Moringa oleifera* (8%) [226], chitosan oligosaccharide (30 mg/kg) [227], inulin (1.6%) [228] and polyphenols (200 to 300 mg/kg) [229]. These supplements have been shown to increase the activity of SOD, glutathione peroxidase (GSH-Px), and catalase (CAT), reduce reactive oxygen species (ROS), and potentially improve the antioxidant capacity of the litter. Oligosaccharides, mainly present in soluble fibers, reduce the colonization of harmful microorganisms in the intestine [230] and increase SCFA production [228]. SCFAs, especially butyric acid, have been reported to decrease oxidative stress in humans [154,231].

Beyond plant-derived ingredients, organic selenium can build up a reserve of Se-Met in tissues, mainly in muscles, which can be mobilized under stressful conditions to improve antioxidant potential [222]. A meta-analysis indicated that sows supplemented with organic selenium (i.e., Se-enriched yeast in doses 0.3 to 0.5 mg/kg) during gestation improved Se content in their serum, colostrum, and milk, as well as GSH-Px activity [232]. However, high doses of selenium can interfere with insulin homeostasis in finishing pigs [233], and while gestating sows have a higher selenium requirement due to placental Se transfer to piglets [222], further studies are necessary to determine the impact of different selenium doses on sows’ longevity and productivity.

Several vitamins can also improve antioxidant status. Supplementing vitamin E (250 IU/kg) from the 107th day of gestation to weaning improved sows’ antioxidant capacity and piglet weaning weight [234]. Vitamin E and polyphenol supplementation increases GSH-Px and SOD activities, and upregulates serum retinol levels [233]. Vitamin C also helps remove excess ROS [222,235]. The combined addition of N-carbamylglutamate (0.05%) and vitamin C (0.05%) from the last third of gestation until weaning has been reported to reduce serum MDA and cortisol, increase IgG levels, alleviate heat stress [236], and improve the birth weight of piglets [235]. However, vitamin supplementation only during the peripartum period may be insufficient to yield positive effects, with longer periods being necessary to achieve optimal results [222].

Beta-carotene [237], essentials oils [238], functional amino acids, and their bioactive precursors such as nitric oxide (NO), polyamines, and glutathione can also be used to prevent oxidative stress. Arginine, for example, is a NO precursor, and cysteine, taurine, glutamate, and glycine are part of GSH synthesis [222,239]. Oregano essential oils (15 mg/kg) modulate the microbiome by increasing Lactobacilli and reducing *Enterococcus* and *E. coli* in sows’ feces, and also increase antioxidant capacity and reduce inflammation in sows during lactation [238]. Sows under oxidative stress have a higher relative abundance of *Phascolarctobacterium* and *Streptococcus*, and their litters have a reduced weight at 21 days, whereas *Bacteroides* is associated with higher litter weights [240]. In addition, healthy sows exhibit a better antioxidant profile in their milk compared to those with PPDS [241].

A wide range of natural extracts, essential oils, and other compounds can be used to reduce oxidative stress with feasibility for application depending on the resources available in each country. Additionally, the complexity of oxidative stress warrants further studies to determine physiological ranges and optimal antioxidant supplementation [239].

### 4.3. Crude Protein and Fat, and Their Impact on Heat Stress

In tropical climates, heat stress represents a critical challenge for pig production, adversely impacting animal health, welfare, and performance, while contributing to considerable economic losses. [242]. Temperatures above 20 °C dramatically increase respiratory frequency and body temperature, causing additional cardiovascular stress [243]. Sows’ cardiovascular systems are frail, with a reduced cardiac output and stroke volume for their BW [244]. This is probably the reason why studies report increased mortality during summer and/or hot days, with heart failure as a major cause of death [12].

Genetic selection has resulted in modern sows experiencing elevated metabolic demands, which are further intensified during the peripartum period and lactation. These heightened metabolic rates lead to increased heart and respiratory frequencies, generating metabolic heat and increasing susceptibility to heat stress [240]. Heat stress not only suppresses feed intake but also induces profound metabolic alterations, including disruptions in insulin secretion patterns, elevated non-esterified fatty acid production, altered microbiome composition, and compromised gut barrier integrity, particularly with increased permeability to lipopolysaccharides. Furthermore, heat stress exacerbates oxidative stress [148,241]. These metabolic disturbances can significantly compromise sow health, especially during the peripartum period [136,245,246].

Several nutritional strategies to alleviate heat stress in sows are being studied. One possible strategy is to reduce CP levels during critical periods such as lactation. Zhang et al. [247] tested CP levels ranging from 19% to 14% (0.90% Lys for both diets), and supplemented amino acids to approximate the ideal profile. The lower-protein diets produced less heat, and sows demonstrated fewer physiological signs of stress and were more productive. However, in this study, lowering CP resulted in higher catabolism, which has not been consistently observed by other authors [248]. By substituting a high-CP diet with a lower one with amino acid supplementation, it is also possible to favorably modulate the microbiome [249], enhance intestinal immunity, reduce oxidative stress, and stimulate enterocyte proliferation [136].

Fat supplementation offers another viable strategy as an alternative energy source, given its lower heat increment compared to other nutrients [250]. Under tropical conditions, 2% fat supplementation has been shown to increase feed and energy intake, improving productivity and milk fat content [250]. Incorporating polyunsaturated fatty acids (PUFAs) and medium-chain fatty acids (MCFAs) can additionally reduce inflammation after labor [251], and help with gut integrity and microbiome modulation, respectively [252,253]. MCFAs and other OAs have antimicrobial activity [252,253]. PUFAs include arachidonic acid (ARA; n-6), eicosapentaenoic acid (EPA; n-3), and docosahexaenoic acid (DHA; n-3), and are involved in eicosanoid production [251]. While n-3 PUFAs are anti-inflammatory, n-6 PUFAs are pro-inflammatory. Both compete for enzymes like COX and LOX during synthesis [254]. Despite their pro-inflammatory role, n-6 PUFAs are essential for biological functions, such as triggering farrowing [255].

The findings suggest that substituting high CP levels for a more precise functional amino acid supplementation can possibly reduce heat stress while maximizing productivity and longevity. On the fat content side, although PUFA use is potentially positive, there are no current feeding recommendations available in swine nutrition guidelines. Given this, further metabolic studies are needed to evaluate the effects of including different PUFAs and determine the optimal n-3/n-6 ratio required to enhance sows’ health and productivity [251].

### 4.4. Methyl Donors and Sows’ Health

Methyl donor compounds are responsible for transmethylation and remethylation, which consist of the transfer of a methyl (–CH3) group from the activated form of MET (S-adenosylmethionine; SAM) to a recipient molecule and for methionine restoration, respectively [256]. Transmethylation is required in at least 100 different metabolic reactions [256,257]. A deficiency in methyl donors promotes inflammation, because homocysteine, an intermediate molecule, is not remethylated, exerting a plethora of adverse effects [256,257,258]. Oxidative stress also inhibits remethylation enzymes and MET adenosyl transferases, increasing trans-sulfuration, which further depletes methyl donor groups [256].

During heat stress, blood flow is redistributed towards the periphery and away from the intestine, which compromises gut integrity, increases endotoxin absorption, and leads to systemic inflammation [256,259]. Methyl donor groups have the potential to inhibit TNF expression by increasing AMPK and to promote gut health and integrity [256].

Supplementation with DL-methionine has been shown to linearly increase CAT and GSH-Px levels, and a 20% supplementation above the requirement increased BW and improved intestinal morphology in growing pigs [260]. While this study was conducted in growing pigs, the results are promising and may be extrapolated to sows during specific phases. In broilers, the deleterious effects of heat stress were ameliorated by Met supplementation [261].

A supplementation of 0.2% betaine, a methionine precursor, during lactation did not improve sow performance during non-summer months, whilst in the summer, the betaine improved subsequent reproductive performance [262]. During gestation, a daily intake of 7.6–9.0 g of betaine in the summer improved piglet birth weight and tended to reduce post-farrowing catabolism [263]. Additionally, Cabezón et al. [264] found that 0.22% betaine supplementation during lactation reduced rectal temperature in sows under heat stress and improved follicle size after weaning.

He et al. observed that supplementation with methionine (4700 mg/kg), folic acid (16.3 mg/kg), choline (2230 mg/kg), vitamin B12 (0.15 mg/kg), and vitamin B6 (1180 mg/kg) from the last mating until farrowing improved piglet weight [265]. Additionally, this supplementation positively influenced the piglets’ microbiota by increasing the relative abundance of SCFA-producing genera. Similarly, Jin et al. [266] reported that maternal supplementation with methyl donor compounds during gestation enhanced piglet growth performance and improved the profile and expression of IGF-1 protein and its receptor in the liver at birth and slaughter.

Other studies with comparable methyl donor supplementation patterns have demonstrated a reduction in the incidence of intrauterine growth restriction (IUGR) in piglets and increased intestinal lactase and sucrase activity [267]. However, despite these promising outcomes, excessive levels of methyl donors may cause metabolic imbalances and negatively affect growth performance [268]. Further research is needed to identify optimal supplementation levels, taking into account environmental temperature, productivity, and other stress-related factors. Key aspects to consider in peripartum feed formulation, along with suggested levels of antioxidant and methyl donor supplementation, are presented in Table 3.

## 5. Farrowing and Lactation

Farrowing is a brief event, accounting for less than 1% of a sow’s lifespan, yet it plays a critical role in determining her productive and reproductive success, as well as her longevity within the herd [257]. The increase in litter size observed in hyperprolific sows is accompanied by a corresponding rise in farrowing duration. Farrowing durations exceeding 300 min are associated with higher piglet mortality, compromised health during lactation, and reduced subsequent reproductive performance [110,188,269]. In modern swine production systems, it is becoming increasingly common for farrowing durations to exceed 400 min [199]. Such prolonged farrowing adversely affects the sow’s health, physiology, welfare, and lactation performance.

Given these impacts, it is crucial to avoid excessively long farrowing durations and, where possible, reduce them to optimize sows’ longevity. While various management practices aimed at enhancing farrowing supervision and shortening farrowing duration have been extensively studied [188,270,271], recent years have seen growing interest in nutritional strategies [109,200,272].

It is also crucial to emphasize the importance of the transition period for farrowing and lactation performance. Reducing constipation, inflammation, oxidative stress, and heat stress is pivotal for improving farrowing outcomes and overall sows’ health—topics already covered in this review. Therefore, this section focuses exclusively on nutritional strategies specific to the farrowing day and in reducing PPDS. Nutritional strategies spanning both the transition and lactation periods will not be addressed here.

### 5.1. Farrowing

The amount of available energy to the sow at the onset of farrowing can help predict its duration [109]. In addition to the activation of the hormonal cascade that initiates birth, sows require uterine contractions to expel piglets. These contractions depend on the coordinated and rhythmic activity of the myometrium, involuntary contractions of the abdominal muscles, and the opening of the birth canal [273]. This entire process is highly energy-dependent. For example, sows that begin farrowing with arterial glucose concentrations below 4 mmol/L exhibit impaired farrowing kinetics, higher piglet mortality rates, and a greater need for assistance during farrowing [109]. In practice, sows that give birth within three hours after their last meal typically maintain adequate plasma glucose concentrations, whereas those that have not been fed for over six hours experience significantly prolonged farrowing durations [274].

Some studies suggest feeding sows up to eight times a day in the days leading up to parturition to maintain proper glucose concentrations during farrowing [274]. However, this approach is often impractical in many swine operations due to labor, financial, and logistical constraints. Furthermore, increasing feed intake close to farrowing can lead to constipation, causing pain, discomfort, and prolonged farrowing durations [186,188].

To address these challenges, recent studies have explored alternative strategies, such as providing sugar before the onset of farrowing [272]. A more modern approach involves administering high-energy oral supplements containing carbohydrates and glycerol immediately after the onset of farrowing. This method has shown positive effects on farrowing duration and piglet vitality [200]. Despite these advancements, a deeper understanding of the energy metabolism of farrowing sows is needed to develop more effective and practical solutions. Key nutritional aspects to consider in pre-farrowing sows are presented in Table 4.

### 5.2. Lactation and PPDS

PPDS is the major puerperal disease in sows [276]. Physiological states are interconnected; urogenital and mammary gland health are influenced by intestinal health and the microbiome [18]. A lack of dietary fiber has been shown to increase constipation and negatively affect microbiome composition, while dysbiosis can exacerbate constipation and impair farrowing traits [125,186,188]. This interplay highlights the importance of dietary interventions aimed at promoting intestinal health. Constipation, in particular, has systemic effects by increasing the absorption of lipopolysaccharides (LPSs), which trigger inflammation and oxidative stress, elevating the risk of PPDS [125]. Furthermore, LPS has been identified as a suppressor of prolactin secretion, which can impair lactation initiation and maintenance [201].

Controlling BCS, including dietary fiber in prepartum diets, and managing heat stress—along with the potential use of antioxidants—have shown promise in mitigating these issues by alleviating constipation, modulating the microbiome, and reducing the incidence of PPDS [24,136,242]. These aspects were discussed in the previous section; therefore, the focus of this section will be on nutritional strategies specifically aimed at reducing the incidence of PPDS, with an emphasis on enhancing prolactin (PRL) levels.

PRL is a key lactogenic hormone, and its suppression due to systemic inflammation can negatively impact productivity [125,277]. Plant extracts, particularly those with estrogenic or hyperprolactinemic properties, have shown promise in enhancing mammary gland development [36]. Previous studies have documented their effects during grow–finishing phases [36], indicating their potential use in the days leading up to farrowing for sows at risk of developing PPDS. Additionally, administering these extracts post-farrowing to sows diagnosed with PPDS may help restore lactation capacity and support recovery.

For example, the use of the polyphenol soybean isoflavone, a natural active phytoestrogen associated with astragalus polysaccharide (200 mg/kg), improved serum prolactin, growth hormone and insulin-like growth factor 1 contents and improved the antioxidant ability of lactating sows [278]. Similarly, silymarin extract increased prolactin concentrations in female rats [279] and cows [280]. The ingestion of 8 g/day of silymarin led to a 51.8% increase in circulating prolactin concentrations 4 days after the onset of treatment [281]. While these findings suggest potential applications for prepartum sows, there is a significant lack of standardized dosing protocols and research focused specifically on late gestation and its effects on farrowing traits. Further randomized clinical trials aim to establish an intervention protocol for PPDS, with these new integrating strategies that promote intestinal health, optimize prolactin secretion, and reduce systemic inflammation holding significant promise for improving sows’ productivity and longevity. Addressing these gaps through targeted research will enhance our understanding of how these interventions can be most effectively applied in modern swine production systems. Table 5 summarizes the main factors that optimize milk yield and health during lactation. Moreover, one of the most critical considerations for a lactating sow is avoiding excessive weight loss (>10% of body weight), as it negatively impacts productivity. Significant weight loss may indicate that the sow was not adequately prepared during gestation, experienced health issues, or was feed-restricted during lactation. All these factors adversely affect sows’ longevity [24].

Moreover, Table 6 summarizes the recommended levels of energy, CP, SID Lys, CF, calcium, and phosphorus to maximize health and longevity during each productive stage. These recommendations are based on the Rostagno et al. [282] and NRC [19] nutrition manuals, as well as the studies referenced throughout this text.

## 6. Conclusions

Sows’ health and longevity are influenced by various factors, many of which can be addressed through tailored nutritional strategies across different life stages. Key aspects include controlling growth rate, ensuring adequate fat deposition, and achieving optimal weight and backfat thickness at mating. Limb health is also critical for maximizing sows’ well-being and productivity. Feed additives such as probiotics, prebiotics, symbiotics, acidifiers, and others can enhance disease resistance, reduce antimicrobial use, improve digestive efficiency, support intestinal health, and modulate immune responses. Additionally, fiber and coarser diets are effective in preventing gastric ulcers.

While several nutritional strategies and potential biomarkers are linked to improved productivity and health, further research across diverse herds is needed to establish causality and confirm the consistency of these findings. Regarding the microbiome, future studies should prioritize systems biology approaches to investigate microbial–microbial and microbial–host interactions.

In conclusion, although numerous nutritional strategies show promise for enhancing sows’ health and longevity, continued research is vital to fully understand their interactions, optimize supplement dosages, and validate their long-term benefits. Integrating these strategies will be essential to realizing the full productive potential of modern sow genotypes.

## Figures and Tables

**Table 1:** Key factors during the rearing period that should be adapted to maximize sow longevity.

Period	Recommendation	Target Aim or Nutritional Intervention	References
Rearing to mating	- Feed gilts separately from growing/fattening pigs;	At mating: - ADG: 600–800 g; - BW: 135–160 Kg; - BFT: 15–20 mm; - Age: 180–220 days; - Estrus: Second or third.	[11,24,31,34,36,84,85]
	- Promote fat deposition over protein deposition;		
	- Reduce dietary protein; - Avoid restricting energy intake during mammogenesis (from 90 days of age to puberty).		
	- Include fiber in diets to reduce gastric ulcers, improve gut health and metabolism, and manage growth.	- CF: 5–10%. For 14 days prior to mating 8–12% (mainly soluble fiber)	[24,86]
	- Supplement calcium and phosphorus to improve limb health.	- 8% above NRC recommendations	[11,54]
	- Supplement trace minerals (Zn, Mn, Cu) and biotin to improve hoof health.	- 50 mg/kg Zn–methionine, 20 mg/kg Mn–methionine, and 10 mg/kg Cu–methionine and 350 μg/kg of biotin	[58,64,65]
	- Reduce gastric ulcer development.	- Avoid pellet diets - Particle size: 700–1000 µm	[75,79,80,82,86]

ADG: average daily gain; BW: body weight; BFT: backfat thickness; CF: crude fiber.

**Table 2:** Nutritional strategies, feed additives, and body condition score during gestation.

Period	Recommendation	Target Aim or Nutritional Intervention	References
Gestation	- Optimize body condition score (BCS)	- BFT: 15–20 mm	[24,92]
	- High-protein diets benefit young sows in recovering BCS - Early gestation is the best opportunity for BCS recovery	- 17 g/day SID Lys to maximize BW gain	[24,31,85,95,98,198]
	- Reduce long fasting periods, gastric emptying, and acid accumulation to prevent gastric ulcers	- Avoid pelleted diets - Particle size: 750–1200 µm - CF: 8–12% (blend of soluble and insoluble fibers) - Increase number of meals for at least two in semi-automatic and automatic systems	[79,80,82,83,86]
	- Minimize aggression behaviors and feed intake discrepancy in group-housed sows	- CF: 10–15% - 220–300% of Try supplementation for 7 days before mixing	[88,111,115,121,122,184]
	- Increase feed intake or provide a specific amino acid profile and energy density in late gestation in undernourished sows	- Use of specific amino acids in the diet, mainly arginine and/or its precursors - 22 g/day SID Lys to maximize lactation performance - Nutrition adjusted according to BCS	[24,97,195]
	- Reduce cystitis	- Use of acidifiers and probiotics	[18,132,133,134,135,140,142,160]
	- Optimize limb health	- Use of trace minerals	[58,64,65]

BFT: backfat thickness; Try: tryptophan; SID; standardized ileal digestible lysine; BCS: body condition score; CF: crude fiber.

**Table 3:** Essential factors in peripartum feed formulation.

Period	Recommendation	Target Aim or Nutritional Intervention	References
Transition	- Reduce constipation, oxidative stress, and inflammation	- CF: 15% (mainly insoluble fibers) - Use of acidifiers and probiotics - Use of antioxidants: - Vitamin E: 150–200 IU/kg - Selenium: 0.3–0.5 mg/kg - Vitamin C: 500 mg/kg of feed - Beta-carotene:15–25 mg/kg - Polyphenols (e.g., resveratrol): 1000–3000 mg/kg of feed - Use of essential oils - Use of functional amino acids (cysteine, taurine, glutamate, and glycine are part of GSH synthesis, and arginine is NO precursor).	[18,24,123,125,132,133,134,135,140,142,160,218,232,234,235,236,237,239]
	- Optimize calcium, phosphorus, and other electrolytes’ metabolism	- Use of negative DCAD diets	[161,203,212]
	- Minimize heat stress	- 2% of fat in the diet - Reduce crude protein - Use of functional amino acids - Use of methyl donors: - Me: 20% higher than requirement - Betaine: 20 mg/kg of feed - Folate: 16 mg/kg of feed - Choline: 2.230 mg/kg of feed	[242,247,250,256,260,262,264]

CF: crude fiber; NO: nitric oxide; GSH: glutathione peroxidase; DCAD: dietary cation–anion difference; Me: methionine.

**Table 4:** Key nutritional aspects in pre-farrowing sows.

Period	Recommendation	Target Aim or Nutritional Intervention	References
Farrowing	- Reduce fasting periods - Reduce interval between last meal and farrowing onset - Optimize glucose/insulin blood levels	- Farrowing duration: maximum 300 min - Last five days of gestation: - Total feed allowance: maximum 3–5 kg/day - Increase number of feeds to at least three times a day - Extra energy supply on the farrowing day (400 g sugar)	[24,109,199,200,272,275]

**Table 5:** Key factors for optimizing milk yield.

Period	Recommendation	Target Aim or Nutritional Intervention	References
Lactation	Optimize milk yield	- Reduce heat stress (reduce crude protein and increase fat in diets) - Reduce postpartum disorders (invest in transition diets) - Use of antioxidants to reduce oxidative stress - Avoid intense catabolism (higher than 10% of body weight)	[18,24,123,125,132,133,134,135,140,142,160,218,232,234,235,236,237]

**Table 6:** Energy, crude protein, crude fiber, lysine, calcium, and phosphorus recommendations to increase sows’ productivity and longevity.

Period	Age	Recommendations
Rearing	Gilts (63–90 d and 27–50 kg)	EM: 4250–4730 kcal/day CP: 230–260 g CF: 70–140 g SID Lys: 12.5–16 g Total Ca: 10.30 g STTD P: 5.10 g Feed intake estimate: 1400–2000 g ADG estimate: 700–774 g
	Gilts (91–119 d and 50–75 kg)	EM: 7300–8300 kcal/day CP: 290–350 g CF: 100–250 g SID Lys: 15–21 g Total Ca: 13.41 g STTD P: 6.60 g Feed intake estimate: 2000–2500 g ADG estimate: 900–1000 g
	Gilts (120–150 d and 75–100 kg)	EM: 7950–10,500 kcal/day CP: 310–350 g CF: 100–290 g SID Lys: 18–20.5 g Total Ca: 15.2 g STTD P: 7.45 g Feed intake estimate: 2500–2900 g ADG estimate: 900–1100 g
	Gilts (150–200 d and 100–150 kg)	EM: 10,500–11,100 kcal/day CP: 340–400 g CF: 175–315 g SID Lys: 17.5–20 g Total Ca: 15.2 g STTD P: 7.45 g Feed intake estimate: 3300–3700 g ADG estimate: 850–1050 g
Early and mid-gestation (0–90 days)	Gilts	EM: 6670–6800 kcal/day CP: 230–270 g CF: 200–400 g SID Lys: 10.5–13 g SID Arg: 7.5–22 g Total Ca: 12.5–17 g STTD P: 5.40–8 g
	Multiparous	EM: 6500–7500 kcal/day CP: 185–230 g CF: 200–400 g SID Lys: 6.5–11 g SID Arg: 4.5–20 g Total Ca: 9.10–18 g STTD P: 4.00–8.5 g
Late gestation (90–114 d) Transition	Gilts	EM: 7500–8500 kcal/day CP: 350–380 g CF: 200–400 g SID Lys: 17.5– 21 g SID Arg: 17.5–32 g Total Ca: 18.5–20 g STTD P: 8.7 g
	Multiparous	EM: 7700–8700 kcal/day CP: 380–400 g CF: 200–400 g SID Lys: 16.5–19.2 g SID Arg: 16.5–32 g Total Ca: 19–20 g STTD P: 9–10 g
Lactation	First parity	EM: 20,500–21,500 kcal/day CP: 1175–1250 g/day CF: 40–80 g/day SID Lys: 60–70 g/day SID Arg: 60–70 g/day Total Ca: 43–53 g/day STTD P: 20–26 g/day
	Multiparous sows	EM: 21,500–22,500 kcal/day CP: 1200–1275 g/day SID Lys: 74–84 g/day SID Arg: 74–84 g/day Total Ca: 45–55 g/day STTD P: 23–27 g/day

EM: Metabolizable energy; CP: crude protein; CF: crude fiber; Lys: lysine; SID: standardized ileal digestible; STTD: standardized total tract digestibility; ADG: average daily gain.

## Data Availability

Not applicable.
